# A large community outbreak of waterborne giardiasis- delayed detection in a non-endemic urban area

**DOI:** 10.1186/1471-2458-6-141

**Published:** 2006-05-25

**Authors:** Karin Nygård, Barbara Schimmer, Øystein Søbstad, Anna Walde, Ingvar Tveit, Nina Langeland, Trygve Hausken, Preben Aavitsland

**Affiliations:** 1 Department of Infectious Disease Epidemiology, Norwegian Institute of Public Health, Oslo, Norway; 2 European Programme for Intervention Epidemiology Training, Swedish Institute for Infectious Disease Control, Solna, Sweden; 3 Municipal Health Authority, Bergen, Norway; 4 Food Safety Authority, District office of Bergen, Bergen, Norway; 5 Unit for Infectious Diseases and Parasitology, Department of Internal Medicine, Haukeland University Hospital, Bergen, Norway; 6 Institute of Internal Medicine, University of Bergen, Bergen, Norway; 7 Division of Gastroenterology, Department of Internal Medicine, Haukeland University Hospital, Bergen, Norway

## Abstract

**Background:**

Giardia is not endemic in Norway, and more than 90% of reported cases acquire the infection abroad. In late October 2004, an increase in laboratory confirmed cases of giardiasis was reported in the city of Bergen. An investigation was started to determine the source and extent of the outbreak in order to implement control measures.

**Methods:**

Cases were identified through the laboratory conducting giardia diagnostics in the area. All laboratory-confirmed cases were mapped based on address of residence, and attack rates and relative risks were calculated for each water supply zone. A case control study was conducted among people living in the central area of Bergen using age- and sex matched controls randomly selected from the population register.

**Results:**

The outbreak investigation showed that the outbreak started in late August and peaked in early October. A total of 1300 laboratory-confirmed cases were reported. Data from the Norwegian Prescription Database gave an estimate of 2500 cases treated for giardiasis probably linked to the outbreak. There was a predominance of women aged 20–29 years, with few children or elderly. The risk of infection for persons receiving water from the water supply serving Bergen city centre was significantly higher than for those receiving water from other supplies. Leaking sewage pipes combined with insufficient water treatment was the likely cause of the outbreak.

**Conclusion:**

Late detection contributed to the large public health impact of this outbreak. Passive surveillance of laboratory-confirmed cases is not sufficient for timely detection of outbreaks with non-endemic infections.

## Background

*Giardia lamblia *(syn. *intestinalis *or *duodenalis*) is an important cause of gastrointestinal illness throughout the world [1,2]. The most common identified non-human source of infection has been water[3], both through drinking water exposure [4-6] and through recreational exposure in swimming pools or during swimming outdoors [7]. In the USA and UK, giardia has been one of the most common identified causes of waterborne outbreaks [4,5,8].

In Norway (population 4.5 million), giardiasis is mainly considered to be an imported disease. More than 90% of the 300–500 annual cases with information on place of infection have been acquired abroad. Most cases are detected at immigrant screening [9]. The domestic cases are mainly caused by secondary transmission from imported cases or sexual transmission among homosexual men, however for some the source is unknown and contaminated water has been suspected [9]. We know of no previous waterborne giardiasis outbreak in Norway [10].

Bergen, located in Hordaland County, is the second largest city in Norway with a population of around 240,000. On October 29^th ^2004, the municipal medical officer in Bergen was alerted by the university hospital to an increase of patients diagnosed with giardiasis. During the last two weeks there had been 27 laboratory confirmed cases among persons with unknown or no travel history. The cases were mainly young adults living in the central part of the city. At the same time, some general practitioners had also reported an increase in consultations for gastroenteritis. In previous years, only 1 – 2 domestic cases of giardiasis were reported annually in Bergen.

An outbreak team with representatives from the municipal health authorities, the local food safety authority, and the water and sewage authorities initiated an outbreak investigation. The objectives were to describe the outbreak, to identify the source, and to implement short- and long-term control measures. We describe here the results of investigation and highlight the problem of timely detection and recognition of outbreaks of giardiasis in non-endemic countries.

## Methods

### Epidemiological investigations

We defined a case of outbreak associated giardiasis as a person who had a stool sample positive for *Giardia *after September 1, 2004, who had visited or stayed in Bergen during the incubation period for giardiasis (defined as one month) and who had not travelled to a highly endemic area for giardiasis during this time.

Every day we obtained information about all new laboratory confirmed cases of giardiasis from the laboratory of parasitology at the university hospital. General practitioners in the area were informed about the outbreak, and encouraged to submit samples for parasitological examination from patients with symptoms consistent with giardiasis. We interviewed the first diagnosed cases by telephone with trawling questionnaires to identify date of illness onset and any common exposures. Throughout the outbreak all newly diagnosed cases were interviewed in order to collect basic information on age, sex, place of residence, travel history, date of illness onset and probable source of infection.

We used a map of the six water supply zones serving the city and their number of recipients, to map the place of residence of each case and calculated attack rates and risk ratio per water supply zone with 95% confidence intervals.

To assess the number of persons requiring treatment during the outbreak, we received data from the newly established Norwegian Prescription Database (NorPD) on number of prescriptions of metronidazol delivered from pharmacies to persons in Hordaland County during January 1^st ^2004 to August 31^st ^2005. NorPD is a national health register containing information on delivery of medicines from pharmacies in Norway, however indications for treatment is not included in the register. Metronidazole is normally prescribed for a variety of indications such as bacterial vaginitis, gingivitis, part of the combination treatment for *H. pylori*, infections with *Clostridium difficile *and other anaerobic bacteria, amoebiasis, giardiasis, profylactically for colorectal surgery. The NorPD was established January 1^st ^2004, so the average monthly number of prescriptions during January 1^st ^to August 31^st ^2004 was therefore used as the baseline, and the excess for the period September 1^st ^2004 to February 1^st ^2005 was assumed to be prescriptions to patients associated with the giardia outbreak.

#### Case control study

The case-control study was restricted to cases living within the central water supply zone and who were known to us by November 7^th ^2004. From the city's population register we chose controls from the same area, individually matched by sex and birth date. Potential controls were contacted by telephone until we had two controls interviewed per case.

Both cases and matched controls were asked about the exposures in the same period; two weeks before symptom onset for the case. Cases and controls that had travelled to a highly endemic country for giardiasis during the incubation period were excluded. The information was collected by telephone interviews using a structured questionnaire targeted to exposures derived from the trawling interviews, including food and drinks consumed different activities, clinical illness, use of health services and treatment.

#### Data analysis

Data were entered and analysed with Epi Info software, version 3.3 (CDC, Atlanta, GA, USA) and STATA 8.0 (Stata Corporation, College Station, TX, USA). For the case-control study univariate analysis of each exposure was conducted by using the procedure for conditional logistic regression for matched case-control data in STATA. Significant variables (p < 0.1) were included in a multivariable analysis using the same regression procedure.

The results are reported as matched odds ratios with 95 % confidence intervals and two-tailed p-values. To assess risk of giardiasis associated with quantity of water consumed, we later performed a group matched analysis including interviewed cases for whom we did not interview individually matched controls. Group matching was based on gender and 10-year age groups.

Investigations of outbreaks are regulated by the Infectious Disease Control Act and regulations in Norway. The investigation to identify the source and implementing control measures is considered as an urgent public health task. Due to this, the investigation is excepted from the requirement of approval from ethical review board. This is in agreement with the International Guidelines for Ethical Review of Epidemiological Studies by the Council for International Organisations of Medical Sciences (CIOMS) (1991).

### Parasitological investigations

All stool samples were examined at the laboratory of parasitology at the university hospital, initially by microscopy. Due to the large increase of stool test requests in the beginning of November, the laboratory decided on November 9^th ^to start using a rapid immunoassay test (ImmunoCard STAT! *Cryptosporidium*/*Giardia *rapid assay; Meridian Bioscience).

### Environmental investigations

The municipality of Bergen is supplied with drinking water from six different waterworks, all using surface water sources. The waterwork serving the central part of Bergen (waterwork A) is interconnected to another water supply (waterwork B) serving the higher parts of Bergen. Some people located in between these water supply zones may in period receive water from the other supply. Together waterwork A and B serve 48.000 people, in addition to restaurants, hotels, offices, universities, hospitals and other facilities located in the central part of Bergen.

Available routine water quality testing results from August to November 2004 were reviewed and compared with results from 2003. The parameters investigated were turbidity, total bacterial count and counts of thermotolerant coliform bacteria, *E. coli *and *Clostridium perfringens *spores.

Starting November 3^rd^, the water and sewage authorities surveyed the catchment area to identify possible sources of contamination of the water supplies, focusing on sewage contamination from residential areas or from a restaurant located nearby the lake. In addition possible contamination from grazing animals or recreational activity was also investigated. Water samples were collected from different locations in the water source and from several small streams that went into the lake. On the 7^th ^and 11^th ^of November, seven parallel samples were taken and investigated for presence of giardia cysts at the Norwegian Veterinary School and the Swedish Institute for Infectious Disease Control.

## Results

### Epidemiological investigation

**Figure 1:**
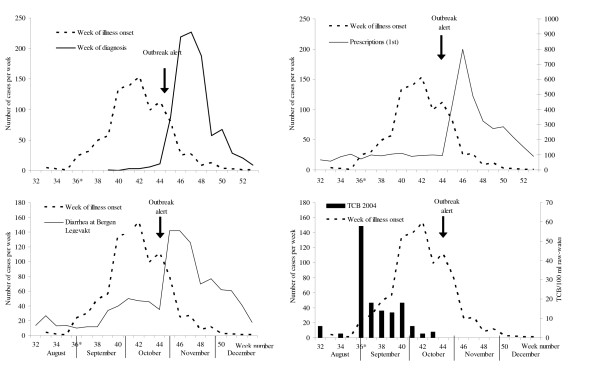
Cases of giardiasis by week of illness onset, Bergen municipality 1/9/2004 – 1/2/2005 (n = 963) and A. cases of giardiasis by week of diagnosis. B. prescriptions of metronidazol delivered from pharmacies to persons in Hordaland per week. C. number of consultations for diarrhoeal illness per week at Bergen legevakt. D. Termotolerant coliform bakteria (TCB) in raw water samples from watersupply A.

The first cases fell ill in the end of August, 2004. Afterwards the number of cases increased gradually, and peaked in middle of October (week 42) (Figure 1). Thereafter the number of cases decreased gradually, and from the middle of November only a few persons fell ill. Consultations for infectious gastroenteritis started to increase at Bergen emergency hospital in the end of September (week 39) (Figure 1c). The outbreak was recognized by the municipal health authorities on October 29^th ^(week 44). Between September 1^st ^2004 and February 1^st ^2005, a total of 1268 lab-confirmed cases were reported.

**Figure 2:**
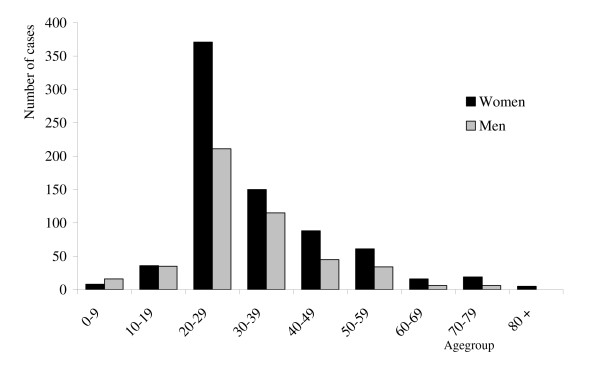
Age- and sex-distribution, giardiasis in Bergen municipality 1/9/2004 – 1/2/2005 (n = 1222).

There was a predominance of adults in the 20–29-year age-group, representing 47 % of the cases, with few cases in children or the elderly. Only 12 cases (1%) were children under 5 years. The female to male ratio was 1.6:1 (Figure 2)

**Figure 3:**
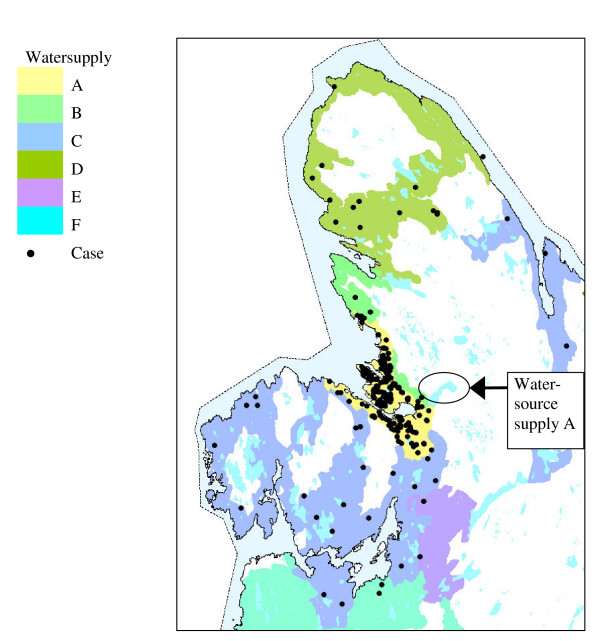
Map of giardiasis cases in Bergen municipality from 1/9 to 1/12 by address of residence and water supply zone (map from water and sewage authority, Bergen municipality).

Of the first 795 cases registered by December 1^st^, 637 cases (80%) lived in the central part of the city, served by water supply A. During the months August to November, a total of 42,774 people received water from supply A. This yielded an attack rate in this supply zone of 149/10,000, compared to 8/10,000 in the other supply zones combined; RR 18 (95% CI: 15 – 22) (Table 1 and Figure 3).

#### Case control study

A total of 27 cases and 54 controls were included in the case-control study. Several exposures were associated with illness in the univariate analysis (Table 2). In multivariable analysis only drinking more than 5 glasses tap water at home (OR 5.9, 95% CI 1.7 – 21) or at a gym located in the city centre (OR 7.2, 95% CI 1.0 – 51) were independently associated with giardiasis. Group matched analysis confirmed the results that cases were more likely to drink a large amount of tap water at home than controls (Table 3).

#### Clinical presentation and treatment

We interviewed 137 cases about clinical symptoms and treatment. Diarrhoea, nausea, stomach pains, flatulence and foul smelling stool were reported by more than 90% of the cases, while vomiting and fever was less common (reported by 36% and 17% respectively). Eighty-three percent of the cases (67 of 81 respondents) reported weight loss, with an average of 5.1 kg (range 1 to 23 kg). Symptoms lasted for a median 30 days range 2–60 days (Table 4).

It took on average 17 days from onset of illness to the first physician contact, and 33 days from illness onset to start of treatment for giardiasis. The cases contacted a physician on average 4.4 times (Table 4). Of 52 cases that became ill during September and October and gave information on date of starting treatment, only 2 cases (4%) started treatment before the outbreak was reported on November 3^rd^. Only six of 83 respondents (7%) were hospitalized.

During the period September 1^st ^2004 and February 1^st ^2005, pharmacies in the Bergen area filled 5700 prescriptions for metronidazol to 4470 different persons. This is an excess of 2500 persons treated in this period, subtracting the monthly average for January 1^st ^to August 31^st ^2004.

### Environmental investigations

Water supply A takes water from a lake located 1–2 km from the centre of Bergen (Figure 3). The catchment area is used for recreational activity, grazing of sheep and there are also some residential areas located close to the water intake. There are no water-animals (i.e beavers) in the area. There are two water intakes at 12 and 17 metres depths respectively. The water is disinfected with chlorine.

Routine samples from water supply A showed high amount of thermotolerant coliform bacteria and *E. coli *in raw water in late August and September, with the highest values in samples taken on August 31 (week 36) (64 *E. coli */100 mL). This was considered to be common during that time of the year. Treated water samples were in accordance with the regulatory requirements for the whole period August – November, with the exception of presence of low levels of *E. coli *in two samples taken on September 14^th ^(week 38)(1 – 2 colony forming units [CFU]/100 mL). This was caused by a short failure in chlorination during the night to September 14^th^, and repeated samples were negative. Turbidity levels in the raw water were within acceptable limits for the whole period (< 1 Formazin Nephelometric Unit).

Due to presence of *Clostridium perfringens *spores in routine samples of treated water in September (1 CFU/100 mL), a sample was investigated for presence of giardia and cryptosporidium (oo)cysts on September 28, 2004. The sample showed 1 presumptive giardia cyst/10 litre. This was interpreted to be in accordance with previous reports of raw water sources in Norway, and considered too low to be of any public health risk. Samples of treated water from November 3, showed five presumptive giardia cysts per 10 l. The raw water samples taken in November showed a maximum of five presumptive cysts per 10 l, varying from zero to five in the parallel samples taken on the same date.

Inspection of the sewage system in the nearby residential area found that the sewage pipes were old with signs of leakage. In times with heavy rainfall, overflow of the pipes would lead to leakage into the lake serving water supply A.

### Control measures

Based on the epidemiological findings together with results of water samples taken from treated water on November 3^rd^, a boil water notice was issued on November 5^th ^to persons receiving drinking water from water supply A. Specific instructions were also issued to hotels, restaurants, other retail food outlets and institutions regarding water use and preventive measures. At the same time the waterworks redirected water from other water supplies, so the area receiving water from water supply A was restricted (Figure 3). From November 5^th ^to November 18^th^, the number of persons receiving water from water supply A was thus reduced from 25,000 to 6,700. A comprehensive mapping system available on the municipality's website was used to inform the public in the affected area about when they could start drinking tap water at their home address. The entire distribution system in the centre of Bergen was flushed in order to remove any remaining Giardia cysts. To stop further contamination, public and private sewage pipes in residential areas and tourist facilities located nearby the water source were checked and improved if needed.

On December 21^st^, 2004 the boiling advice was lifted. This was decided because all recent water samples were negative for Giardia cysts, and all the identified contamination sources had been eliminated. Since many residents of Bergen were still ill, the health authorities strongly advised that the public maintains a high level of personal hygiene and stressed that the public should wash their hands thoroughly after visits to the lavatory. There was a special awareness on possible secondary transmission in child care institutions and swimming pools.

A water treatment plant with filtration and UV disinfection had already been commissioned for the affected water supply before the outbreak, with planned start of operation in 2007. In the interim period, a temporary UV plant was opened in February 2005 to provide additional disinfection for the water supply while the new treatment plant is being built.

## Discussion

We have described the first recognized waterborne outbreak of giardiasis in Norway. This is also one of the largest waterborne outbreaks of any cause reported in Norway during the last decades, with almost 1300 laboratory confirmed cases, and 2500 persons receiving medical treatment. It is estimated that around 48,000 people were exposed to contaminated drinking water during the outbreak. A heavy rainfall during a short period in September may have contributed to this outbreak by overloading the old sewage system, thus causing leakage to the lake. This emphasizes the importance of watershed protection in residential planning in order to diminish the risk of contamination of the water supply from sewage overflow. Norway's drinking water regulations require two independent hygienic barriers between the water source and the customer, of which one can be good source water protection. In the current outbreak, the water supply did not adhere to this requirement since source water protection was insufficient and chlorine disinfection in the doses used does not constitute a hygienic barrier towards protozoan parasites.

Persons who drank a lot of water had a much higher risk of illness. Close to two thirds of the interviewees drank more than 5 glasses of water daily, and many reported drinking several litres daily. To avoid recall bias they were asked explicitly for the period before falling ill, and they stated this was what they normally would drink – many mentioned this as part of dieting, healthy living or exercising. Since many young people, and especially women, drink a lot of water, for these reasons, this might have contributed to the observed age and sex distribution of the cases. In a Norwegian survey from 1997 women and men in the age-group 16–29 years drank on average 390 ml and 323 ml water/day respectively compared to 338 ml and 276 ml for all adult women and men respectively [11]. The low attack rate among children and elderly suggests that the contamination probably has been very small, so that large amounts were needed for infection. The demographic characteristics of the affected area could also have contributed to the observed age-distribution, as it is mainly a business area and with rental accommodations for students.

The epidemic curve indicates that most cases must have been infected during the period from the end of August until the beginning of October. Since Giardia cysts can survive in water for 1–2 months, the contamination may have occurred over a limited period in late August – early September. Several factors may have delayed the detection of the outbreak until the end of October. Many patients delayed seeing their doctor because the symptoms were neither acute nor serious. Thereafter, most physicians did not submit stool samples in patients with mild diarrhoea. If they did, they rarely requested examination for giardia since the patients had not been abroad [12], and the laboratories do not routinely analyze faecal samples for parasites. Many patients contacted their physicians several times without being diagnosed with giardiasis. Thus, the pathogen-specific Norwegian surveillance system for communicable diseases could not detect the outbreak in a timely manner, a recognized problem for laboratory based surveillance systems [13]. Unfortunately, in this outbreak, several other indications that an outbreak was imminent were also overlooked.

Firstly, routine samples from the water supply showed high levels of faecal indicator bacteria and *E. coli *in raw water samples taken in the end of August. However, samples from treated water were in accordance with drinking water requirements, thereby giving a false sense of security. Chlorination will effectively remove indicator bacteria from the water, but chlorine-resistant pathogens such as protozoan parasites can still be present. A survey of surface water in Norway showed that cryptosporidium and giardia were present in 18.5 % of sources sampled, albeit in small numbers (80% of the samples had only 1 cyst/10 l, and the highest concentration observed was 3 cysts/10 l)[14]. Many Norwegian waterworks still rely on chlorination as the only water treatment, and up to now very few domestic infections with these parasites have been diagnosed. With increasing travel to endemic areas, the risk of contamination of water sources may increase, and may lead to a change in the incidence of these infections if measures to prevent waterborne transmission are not taken.

Secondly, several clinicians had noted an increase in consultations for gastrointestinal symptoms. Statistics from Bergen emergency hospital showed an increase in consultations for infectious gastroenteritis already in late September, more than a month before the outbreak was recognized. In the medical microbiological laboratory serving the city, suspicions should have been raised when there was a large increase in requests for stool cultures during September and October. Unfortunately, most clinicians and microbiologists will delay outbreak reporting until a diagnosis is confirmed. There is a need to lower physicians' threshold for reporting unusual events to public health authorities.

Thirdly, the food safety authority was contacted by TV journalist 7–10 days before the outbreak was detected; asking if there was an outbreak going on. The food safety authority contacted the microbiological laboratory at the local hospital, which confirmed that they had received many stool samples, but that all were negative for bacterial and viral pathogens. The municipal health officer was contacted, but he had not received any reports about any suspected outbreak. This was then not followed up further.

Other possible sources for detection of outbreak should be considered in the future in order to reduce delays in recognizing outbreaks. A new electronic surveillance system is currently under development in Norway, based on daily collection from all general practitioners and emergency rooms of number of consultations and their diagnoses. Following a large waterborne outbreak of cryptosporidiosis in Milwaukee, the timeliness and usefulness of the various sources for outbreak detection was evaluated [15]. Water quality data and water customer complaint logs were the most timely, while clinical laboratory data lagged more than two weeks. Other surveillance systems that the authors considered useful included nursing home diarrhoeal rates, hospital emergency room visits, school absentee data, and diagnostic laboratory test data. Due to a limited period of transmission and a delay in clinical consultations in this outbreak, the early warning system that would have been most timely in limiting the extent of the outbreak is the system based on water quality data. Detection based on clinical- or laboratory-based surveillance would probably not had a large effect in limiting the extent of the outbreak, however earlier detection and identification of the aetiology would have caused more timely diagnosis of the patients, earlier start of treatment, less patient suffering and limited secondary transmission.

## Conclusion

We have described a waterborne outbreak caused by contamination of a municipal water supply serving the centre of the second largest city in Norway. Late detection contributed to the huge public health impact of the outbreak. This outbreak highlights the importance of non-complacency regarding provision of safe drinking water, and that questions from media or clinicians regarding suspected outbreaks need to be investigated thoroughly. Relying on disease-specific surveillance systems for detection of outbreaks is not sufficient when the pathogen is difficult to diagnose, or when it is non-endemic in the area and therefore not part of the routine diagnostic workup.

## Competing interests

The author(s) declare that they have no competing interests.

The study was funded by internal funds from the Norwegian Institute of Public Health and the municipality of Bergen.

## Authors' contributions

KN was the principal investigator of the epidemiological investigation of this outbreak; she carried out the statistical analysis of the case-control study, and drafted the manuscript. BS participated in data gathering and analysis, ØS was responsible for leading the investigation, AKW and IT conducted patient interviews and participated in management of the outbreak, NL and TH carried out patient management and treatment and PA participated in the design of the study and helped to draft the manuscript. All authors read and approved the final manuscript.

## Pre-publication history

The pre-publication history for this paper can be accessed here:



## Figures and Tables

**Table 1:** Attack rate of giardiasis by water supply zone, Bergen municipality Aug-Nov 2004 (n = 795)

Watersupply	Cases	Number of recipents	Attack-rate (per 10,000)		
A	637	42,774	148.9		
B	15	9,685	15.5		
C	89	105,440	8.4		
D	33	34,406	9.6		
E	4	14,266	2.8		
F	13	23,848	5.5		

				Risk ratio	95% confidence interval

B+C+D+E+F	158	194,519	8.1	Ref.	
A	637	42,774	148.9	18.3	15.4 – 21.8

**Table 2:** Matched univariate conditional logistic regression analysis of selected dichotomous risk factors among cases of giardiasis and matched controls, water-supply zone A, Bergen municipality 1/9 – 15/11 2004.

	Cases (%) (n = 27)		Controls (%) (n = 54)		Matched OR	95% CI	p-value
Having children in household	8	33%	17	34%	1.2	0.3 – 4.5	0.8
Having dog/cat	8	30%	7	13%	5.3	1.0 – 26.6	0.04
Salad	20	87%	45	90%	0.7	0.2 – 3.3	0.7
Tomato	19	83%	44	85%	0.8	0.2 – 3.2	0.8
Cucumber	20	83%	41	82%	1.2	0.3 – 4.3	0.8
Raw leek	9	38%	9	18%	7.1	0.9 – 58.9	0.1
Mineralwater	11	42%	27	51%	0.6	0.2 – 1.8	0.4
Coffee	18	72%	28	52%	2.4	0.7 – 7.9	0.2
Beer	8	33%	8	15%	3.3	0.9 – 12.7	0.06
Water at home (>5 glass)	20	74%	12	22%	7.3	2.4 – 21.8	<0.01
Water at the gym	10	38%	8	15%	5.2	1.1 – 26	0.03
Water in cafe or restaurant	10	38%	13	25%	1.8	0.6 – 5.2	0.3
Drinking water at work	14	56%	24	45%	1.6	0.6 – 4.4	1.6
Supermarket A	22	88%	27	55%	6.5	1.4 – 29.2	<0.01
Supermarket B	12	52%	19	40%	1.2	0.4 – 3.5	0.7

NOTE. CI, confidence interval; OR, odds ratio.

**Table 3:** Risk of giardiasis associated with quantity of water consumed among residents in water zupply zone A, Bergen municipality 1/9 – 15/11 2004. Group matched analysis by sex and 10-year age groups (83 cases, 54 controls).

Water intake	Cases	%	Controls	%	OR*	95% CI
< 1 glass	1	1 %	4	7 %	Ref	-
1 – 2 glasses	8	10 %	11	20 %	3.2	0.2 – 69.5
3 – 5 glasses	23	28 %	27	50 %	4.8	0.4 – 64.7
more than 5 glasses	51	61 %	12	22 %	7.4	1.2 – 44.5

* chi-square test for linear trend: 19.7; p < 0.001.

**Table 4:** Treatment, severity of disease and time-delays in health care – laboratory-confirmed cases of giardiasis, Bergen 1/9 – 15/11 2004

	Yes	Total	Percentage	Average	Median	Range
Duration (days)	-	82	-	32 d	30	6 – 60
Weight loss	67	81	83%	5.4 kg	5	1 – 23
Physician contacts (visits or phone)	72	73	99%	4.4 times	3	1 – 21
Absence from work/school	54	76	71%	10.5 d	8	1 – 30
Hospitalisation	6	83	7.2%	3.8 d	2.5	1 – 11
Antibiotic treatment	57*	83	69%	-	-	-
Patient delay in contacting health services (days)	-	74	-	17.1 d	16.5	0 – 44
Physician delay in start of treatment (days)	-	47	-	18.0 d	15.5	0 – 54
Total delay between symptom onset and start of treatment (days)	-	51	-	33.1 d	31	9 – 69

* at time of interview (8-11-04)
